# Effect of Nano-Reinforcement Topologies on the Viscoelastic Performance of Carbon Nanotube/Carbon Fiber Hybrid Composites

**DOI:** 10.3390/nano10061213

**Published:** 2020-06-22

**Authors:** Suma Ayyagari, Marwan Al-Haik, Yixin Ren, Dhriti Nepal

**Affiliations:** 1 Department of Aerospace Engineering, Embry-Riddle Aeronautical University, Daytona Beach, FL 32114, USA; ayyagars@my.erau.edu; 2 Air Force Research Laboratory, RXCCP Division, Wright-Patterson AFB, OH 45433, USA; yixin.ren.ctr@us.af.mil (Y.R.); dhriti.nepal.1@us.af.mil (D.N.)

**Keywords:** carbon nanotube, carbon fiber, viscoelastic, damping, glass transition

## Abstract

In this investigation, multi-walled carbon nanotubes (MWCNTs) were grown over carbon fiber fabrics via a relatively nondestructive synthesis technique. The MWCNTs patches were grown into three different topologies: uniform, fine patterned and coarse patterned. Hybrid carbon fiber-reinforced polymer composites (CFRPs) were fabricated based on the patterned reinforcements. Tensile tests, dynamic mechanical thermal analyses (DMTA) and flexure load relaxation tests were carried out to investigate the effect of the patterned nano-reinforcement on the static, dynamic, glass transition, and viscoelastic performance of the hybrid composites. Results revealed that the hybrid composite based on fine-patterned topology achieved better performance over all other configurations as it exhibited about 19% improvement in both the strength and modulus over the reference composite with no MWCNTs. Additionally, the increase in glass transition for this composite was as high as 13%. The damping parameter (tan δ) was improved by 46%. The stress relaxation results underlined the importance of patterned MWCNTs in minimizing the stress decay at elevated temperatures and loading conditions. Utilizing patterned MWCNTs topology significantly reduced the stress decay percentage at the thermomechanical conditions 60 MPa and 75 °C from 16.7% to 7.8%. These improvements are attributed to both the enhanced adhesion and large interface area by placing MWCNTs and by inducing an interlocking mechanism that allows the interaction of the three constituents in load transfer, crack deflection and hindering undesired viscoelastic deformations under different thermomechanical loadings.

## 1. Introduction

Fiber-reinforced plastics (FRPs), composites based on high strength and stiffness fibers (e.g., glass and carbon), have long been utilized for structural applications due to their outstanding mechanical properties [1]. However, the expansion of applications of FRPs to environments prone to elevated temperatures makes it crucial to investigate their rheological properties. It is important that the FRPs exhibit thermal stability and resistance to elevated temperatures. While there are several techniques to evaluate the thermal stability of polymers and their composites, such as differential scanning calorimetry (DSC), thermo-mechanical analysis (TMA), thermogravimetric (TGA) and dynamic mechanical thermal (DMTA) analyses, DMTA is most suitable for FRPs as it is sensitive enough to capture the macro and microscale processes and molecular relaxations of the polymeric matrices [2,3]. The DMTA is employed mostly to probe the glass transition temperature (Tg) of polymers and their composites. The glass transition is the most crucial physical transition in polymers, and it occurs when the chains of a polymer gain enough energy (e.g., from thermal sources) to overcome the energy barriers for bond rotation. Hence, the polymer goes from a frozen glasslike condition with limited mobility to a mobile system that achieves thermodynamic equilibrium [4]. The glass transition temperature is manifested by a drastic drop in the storage modulus associated with this change, typically by several orders of magnitude for an amorphous polymer [5]. T_g_ is also identified by an increase in the values of loss modulus and internal friction tangent (tanδ) [6]. The DMTA is valid for several polymeric systems including thermoplastics, thermosets and has been utilized to characterize various polymeric composites [7,8].

While the FRPs’ thermal stability can be characterized by their glass transition, their thermo-mechanical performance is manifested by viscoelastic behaviors such as creep and stress relaxation [9,10,11,12,13,14,15]. Polymer matrix composites are prone to growing deformation under constant loads (creep) and tend to carry less load under constant displacement (stress relaxation) in relatively long time periods. These behaviors are accelerated under elevated temperatures environments. If not accounted for, these time-dependent behaviors can jeopardize the load-bearing capability of a composite structure, leading to premature failure or structural instabilities. Like many other mechanical properties, the creep compliance and stress relaxation modulus (as two governing material parameters for viscoelasticity) are much weaker along the transverse directions to the fiber orientation [16]. This heightens the importance of studying the flexural viscoelastic properties in FRPs where the axial direction is designated as the fiber direction which exhibits the least deformation imposed by time or temperature variations. Flexural creep or load relaxation tests for thin, layered FRPs can be carried out with a three-point bending fixture in DMTA instruments. Application of DMTA as one of the most convenient load displacement-controlled testing tools are practiced extensively both for static and dynamic analyses of polymeric materials. Studying the viscoelastic behavior of polymeric composites in general and creep and stress relaxation, particularly utilizing DMTA, has been reported [17].

The root cause of the viscoelastic deformations in FRPs is the rheological nature of the polymeric matrix where the constituent molecular chains tend to expand under thermomechanical loads by sliding against each other, unfolding and straightening [18]. These molecular scale movements add up to a macroscale measurable creep strain or stress relaxation. The addition of carbon fibers enhances the mechanical properties of the polymers considerably and enhances their resistance to viscoelastic deformation. Nevertheless, the stretching of the fibers from their initial waviness and the slippage mechanisms at the interface between the fibers and matrix provide other sources for viscoelastic deformations in FRPs [19].

To hinder these slippages, the FRPs can be retrofitted by adding rigid nano fillers to the polymer matrix [15]. For nanofillers with considerable van der Waals type forces, such as carbon nanotubes (CNTs), the percolation limit is of a profound importance. Agglomerated CNTs are not only poorly adhered to the matrix, but also give rise to stress concentration compromising the effect of the CNTs as reinforcement. Several investigations employed shear mixing, ball milling [20,21] ultrasonication [22], clandering [23] and external fields to properly disperse the CNTs in polymeric matrices [11,24]. While some of these techniques lessen the severity of agglomeration, most are not effective beyond ∼3 wt.% of CNTs [25,26].

In lieu of direct mixing of nanophase, generating well-attached, nanoscale physical obstacles on the fibers (whiskerization) can eliminate the dispersion and agglomeration problems. The growth of CNTs on commercially available carbon fibers has been demonstrated earlier [27,28]. Zhang et al. [29] have grown high density multiwall carbon nanotubes (MWCNTs) using the catalytic chemical vapor deposition (CCVD) process at high temperatures ~800 °C on the surface of carbon fibers. The results revealed a 40% decrease in the tensile strength. In several of our pervious investigations, we have shown that growing CNTs using a nondestructive technique; graphitic structures by design (GSD) can improve the strength, damping parameter, fatigue resistance and interlaminar fracture toughness of FRPs [30,31,32]. We also have shown that growing the CNTs in patterned topology rather than uniform growth enhances the interlaminar shear strength [33].

Several investigations employed growing CNTs randomly over carbon fiber toward improving the viscoelastic response. Miyagawa et al. [34] investigated a hybrid epoxy nanocomposites reinforced by fluorinated single wall carbon nanotubes (FSWCNT) and vapor-grown carbon fibers (VGCF). They observed a reduction of T_g_ by 30 °C and 20% improvements of the storage modulus. The authors reported that the improvements were mostly attributed to adjusting the amount of the curing agent. Sharma et al. [35] attempted to improve the interfacial bonding by coating coiled carbon nanotubes on carbon fiber surface through a single-step chemical vapor deposition (CVD) process. They observed that the T_g_ increases by 12.5%, while the damping factor (tan δ) dropped by 11.5% at 30 °C due to the addition of coiled CNTs to the fiber surface. Wei and Gao [36] investigated the viscoelastic behavior of high-performance unidirectional polymer-based hybrid composites in which graphite fibers are embedded into the epoxy matrix enriched by CNTs. They reported that the transverse creep performance of unidirectional hybrid composites can be improved significantly by adding the CNTs. However, they reported no improvements along the fiber direction and negligible changes on the other mechanical properties along the longitudinal direction.

Although CNT significantly improves the composite viscoelastic properties, there is still a gap in understanding the effect of interphase topology, especially the role of a tailored interface between CNT and carbon fiber. Additionally, virtually no attempt was made to “tailor” the interface between the CNTs and both the carbon fiber and the polymer matrix. Most of the previous work focused on functionalizing the fiber or the matrix via chemical means.

The goal of this investigation is to probe the effects of growing CNTs into different topological patterns on the composites’ rheological properties including the T_g_ and damping. The investigation also characterizes the elastic (tensile) and viscoelastic (load relaxation) performances of composites with different CNTs topologies.

## 2. Materials and Experimental Methods

### 2.1. Synthesis of Hybrid Reinforcements

PAN-based carbon fibers with 3k bundles Thornel^®^ T650 (Cytec, Inc., Mount Pleasant, TN, USA) were utilized as the reinforcement throughout this investigation. The sizing on the fibers was removed by placing the as received sized fibers in a tube furnace at 550 °C for 30 min under inert environment (nitrogen). The MWCNTs growth process was performed following the graphitic structures by design (GSD) protocol explained in detail elsewhere [31,37]. The GSD synthesis technique requires pre-deposition of a catalyst metal (i.e., nickel in this study) on the surface of the carbon fibers. The CNT’s growth initiates at the areas where the catalyst material is pre-deposited. For growing uniform CNTs forests over the carbon fiber fabrics, a uniform layer of nickel was deposited. An ATC Orion (AJA international Inc., Scituate, MA, USA) high-vacuum sputtering system was utilized to deposit a 10.0-nm-thick layer of nickel on both sides of the square carbon fabric samples under 3 milli torr pressure of argon gas where 100 W of power was supplied to the sputtering target. To achieve a checkerboard patterned CNTs forests over the carbon fiber fabrics, two perforated polyester mesh templates were utilized (Components Supply Co Inc., Fort Meade, FL, USA) with mesh openings of 105 and 53 μm, thread diameters of 70 and 31 μm, and open area percentages of 33% and 40%, respectively.

The GSD-CNTs growth process was carried out inside a quartz tube reactor equipped with a thermal controller and three-input gas mass flow controllers. The process starts with a reduction step, under a H_2_/N_2_ gas mixture atmosphere at 550 °C for 2 h. Then, the process is followed by the flushing step in which the tube reactor is flushed with N_2_ gas to get rid of any residuals of the previous step. Subsequently, the CNTs growth step begins, maintaining the constant temperature at 550 °C for 1 h under a C_2_H_4_/H_2_/N_2_ environment.

### 2.2. Composites Fabrication

Four composite configurations were fabricated: One with uniform growth of CNTs as a result of performing the GSD growth on a sample pre-sputtered with uniform catalyst film, the second sample with a catalyst patterned with a polyester mesh opening of 105 micron on carbon fabric, the third with a catalyst patterned with a polyester mesh opening of 53 micron on carbon fabric, and the fourth based on a de-sized carbon fabric referred to as reference sample. Each composite comprised two plies adhered by an epoxy matrix; Aeropoxy^TM^ (PTM&W Industries, Inc., Santa Fe Springs, CA, USA). This epoxy is a mixture of PR2032, a Bisphenol-based resin, and PH3660, a hardener. This resin is specially designed for structural production applications with a medium viscosity of 1650 cPs at room temperature. It aids the lamination process and easily wets out carbon, aramid fibers, and fiberglass. The hardener has a lower viscosity of 190–200 cPs at room temperature. When both the resin and hardener are mixed in the ratio of 100:27 by weight, a viscosity of 800–875cPs and a T_g_ of 91 °C are reported by the manufacturer. The hand-layup method was used for lamination. Following the lay-up of the two plies impregnated with epoxy, it was sealed in a vacuum bag. The four composite laminate configurations were processed using a composite autoclave (Econoclave, ASC Process Systems, Valencia, CA, USA). Guided by ASTM standard D5687 [38], a pressure of 70 psi and a vacuum of 25 torr were maintained in the chamber throughout the curing process. The autoclave cycle had multiple steps: Isothermal step for 1h at 22 °C, heating to 93 °C, isothermal step for 2 h at 93 °C, then cooling down to room temperature.

### 2.3. Mechanical Testing

Preparation of the composites based on the four different fiber configurations followed the ASTM standard D5687. The laminates were cut into 12.50 × 1.25 cm tensile coupons and were tested following the ASTM standard D3039/D3039M-08 using an MTS Criterion™ Model 43 machine (MTS, Inc., Eden Prairie, MN, USA) equipped with a 25.4-mm gauge length extensometer to measure the strain. The tests were conducted under constant crosshead speed of 1.0 mm/min until failure occurred. A minimum of eight samples were tested for each configuration.

A separate batch of specimens was prepared for DMTA. A Discovery Hybrid Rheometer (DHR) with DMTA capabilities (TA Instruments^®^, Co., New Castle, DE, USA) was utilized. The DMTA tests were carried out following the ASTM D7028-07 and ASTM D5023-15 standards. To ensure that the applied loads/strains are within the elastic range, the linear viscoelastic range of the different composite configurations were determined using a method described in our previous work [39]. Composite coupons of size 50.00 mm × 6.25 mm were cut accordingly. A three-point bending fixture with span of 40 mm was used to mount each of the composite samples. In the temperature sweep mode of the DMTA, constant frequencies of 0.1, 1.0, 5.0 and 10.0 Hz were applied, while varying the temperature from 22 to 140 °C at a constant strain of 0.05%. The frequency sweep test was performed with a frequency range from 1 to 80 Hz scanning at room temperature using the same strain applied in the temperature sweep test.

The DHR system was also utilized to conduct flexural load relaxation tests. The load relaxation tests carry the advantage of revealing a multitude of strain rates compared to a single strain rate in a steady creep test [12]. The stress relaxation tests play a major role in revealing the composite materials’ viscoelastic/viscoplastic behaviors [40,41,42,43,44,45]. Stress relaxation tests provide the rate of decrease in stress for any state of stress that results from maintaining a constant strain during uniaxial loading. Flexural load relaxation tests entail loading the sample till reaching the desired stress level then maintaining the corresponding strain constant for 30 min. Based on the linear range of viscoelastic response, stress relaxation tests were carried out at four different strain levels at each temperature. The values of the strain were chosen to correspond to the stress values 15, 30, 45 and 60 MPa, under 25, 50 and 75 °C, respectively. The relaxation tests were carried out up to 75 °C where it is still below the neat epoxy’s reported glass transition (~93 °C) so that unexpected rheological behavior can be evaded.

### 2.4. Morphology Characterization

Light microscopy (Olympus GX35 inverted microscope, Olympus Co., Tokyo, Japan), scanning electron microscopy (SEM, FEI Quanta 650, Thermo Fisher Scientific Co., Waltham, MA, USA and Gemini 500 SEM, Zeiss Co., Berlin, Germany) and transmission electron microscopy (TEM, FEI Talos, Thermo Fisher Scientific Co., Waltham, MA, USA) at an acceleration voltage of 200 kV) were utilized to examine the size and morphology of the grown CNTs and their growth topology.

## 3. Results and Discussion

### 3.1. CNTs Growth Analysis

**Figure 1:**
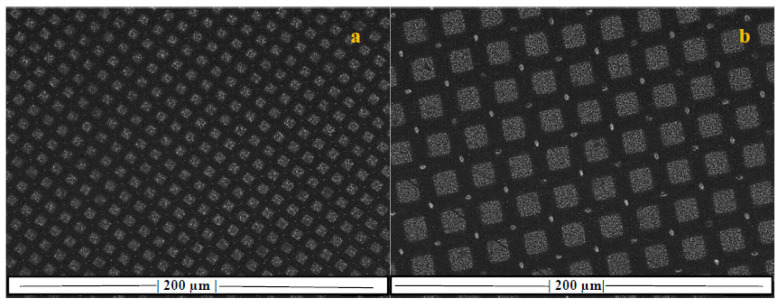
Light microscopy micrograph of the mesh utilized for patterning a (**a**) 53- and (**b**) 105-micron pattern with thin film of Ni (lighter colored area).

**Figure 2:**
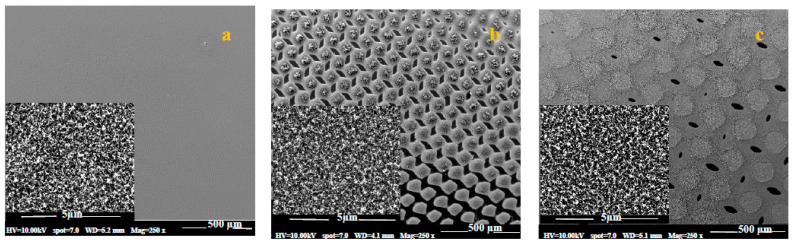
Scanning electron microscopy (SEM) micrographs of (**a**) uniformly grown MWCNTs over silicon wafer at two different magnifications, and (**b**) MWCNT patches grown in a 53-micron checkerboard pattern and (**c**) 105-micron checkerboard pattern over a silicon wafer. Lighter colors indicate MWCNT patches. Main scale 500 μm, in-set scale 5 μm.

The realization of the uniform and patterned growth was conducted by carrying the GSD procedures on a flat silicon wafer after depositing nickel catalyst uniformly or in 53- or 105-micron patterns, as shown in Figure 1. Upon carrying the GSD procedures, the area coated with Ni promoted the growth of CNTs, as shown in the SEM micrographs of Figure 2. The inset figures show that, despite the difference in the growth topology, the CNTs themselves are identical. These patterns were used to grow the CNTs on the carbon fiber fabric.

**Figure 3:**
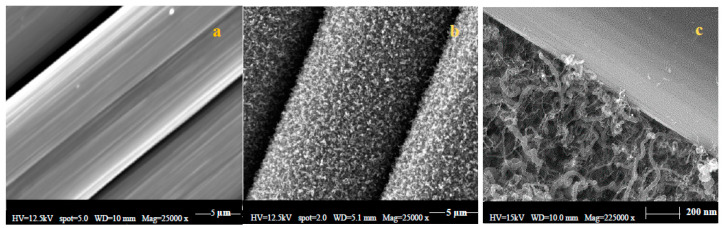
Scanning electron microscopy (SEM) micrographs of (**a**) desized carbon fiber, and (**b**,**c**) GSD grown MWCNTs over carbon fibers at two different magnifications.

The changes on the carbon fibers surface as a result of growing MWCNTs via the GSD method are shown in Figure 3. Two distinct morphologies of CNTs are visible in Figure 3c (captured using high resolution Gemini SEM): whisker-like and spiral filaments. Nearly all the filaments are less than 200 nm in diameter, sub-micron in length, although the distribution of filaments is ‘bimodal’. The morphology of the sub-micron filaments is not uniform. Some clearly have smooth surfaces, whereas others grow in tight spirals or are kinked. The sub-micron filaments are not straight, and hence form a tangled mat.

**Figure 4:**
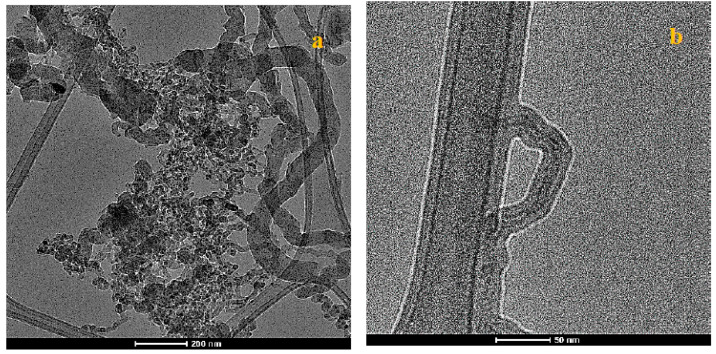
(**a**,**b**) TEM micrographs at different magnifications of the grown carbon species using the GSD technique.

Transmission electron microscopy (TEM) micrographs, as shown in Figure 4a, illustrate the relative diameter (~100 nm) of the larger filaments grown under the GSD process. It can also be seen that the filament growth is tip based due to the metal particle being found at the tip of the filament. Additionally, worth noting is that the heavily entangled filament with the metal particle appears to be hollow with crystalline walls indicating MWCNTs, as shown in Figure 4b with tube diameter within 25 nm.

### 3.2. Tensile Test Results

**Figure 5:**
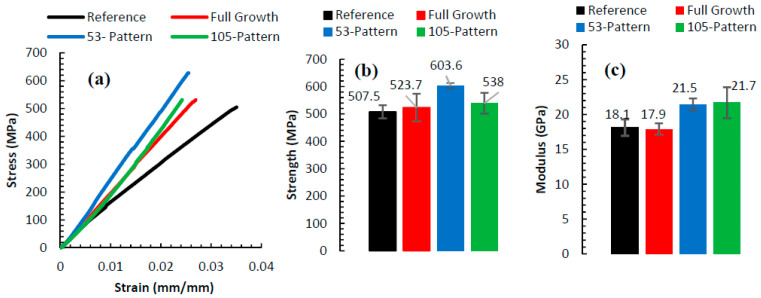
(**a**) Representative tensile tests of the different composites. The average (**b**) tensile strength and (**c**) axial modulus of the different composites. Error bars represent the standard deviation.

Figure 5a shows representative stress–strain curves for the different composites configurations based on the desized (reference) carbon fiber, fibers with uniform growth of MWCNTs and fibers based on the two-patterned growth—the 53 and 105 pattern. All the samples exhibited linear-elastic behavior up to the point of failure. The strength and modulus for the different composites were averaged for eight samples for each configuration. These results are shown in Figure 5b,c. A noticeable observation is that growing CNTs uniformly yielded a miniscule increase in the strength, ~3%, compared to the composites based on desized fibers (reference). The dense growth of the MWCNTs could have limited the impregnation (wetting) of the epoxy into this dense CNT layer to reach the base carbon fiber which is crucial for improving the mechanical properties.

On the other hand, utilizing coarse (105-micron) and fine (53-micron) patterns assisted in resolving the issue of epoxy impregnation. The spacing in-between the MWCNTs patches, as depicted by Figure 2b,c, allowed for forming an interlocking mechanism between the MWCNTs, carbon fiber, and epoxy. Such mechanism assisted in improving the strength by 19% for the fine, and 6% for the coarse patterns, respectively.

It is worth pointing out that, composites based on T650 carbon fibers with surface-grown CNTs using CVD were attempted by another research group [29]. However, it was reported that for the unsized T650 with uniform growth of CNT (grown at 750 °C), the tensile strength and modulus were identical to the composite based on desized fibers; i.e., the growth of CNTs did not induce neither improvements nor deterioration on the composite’s strength. These results agree with our finding for the case of composite with uniform growth of CNTs. Additionally, the 3% improvement on the strength can be attributed in part for utilizing relatively lower temperature in the GSD (550 °C compared to 750 °C in CVD). Thus, one can conclude that the significant improvements encountered are mostly attributed to the patterned growth, especially for the case of the fine pattern of 53 micron. The improvements achieved by the patterned growth were also accompanied by enhancement on the modulus—roughly by 18.7% for both patterned growths. In other words, stronger and stiffer bonding at the matrix/fiber interface leads to a higher elastic modulus. Finally, Figure 5a suggests that the improvements of the strength and modulus gained by the patterned growth of CNTs come at the expense of reduced ductility evident by the reduced strain to failure.

**Figure 6:**
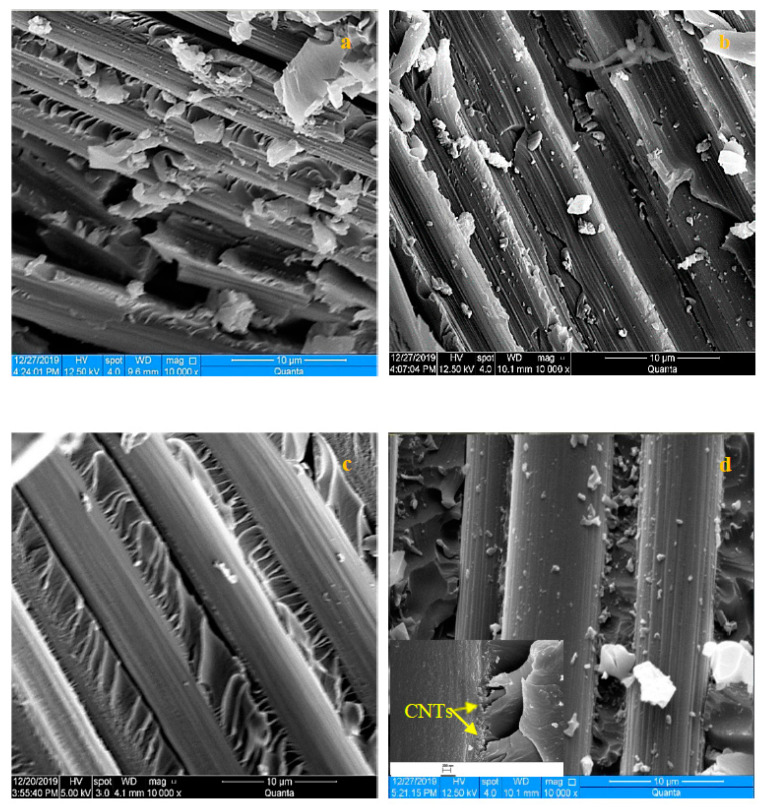
SEM fractography of the composites based on (**a**) reference, (**b**) uniform growth, (**c**) 105-micron patterned growth, and (**d**) 53-micron patterned growth fibers; inset scale bar 200 nm.

To probe the effects of the growth patterns further, we analyzed the fracture surface of the tensile samples. Figure 6a clearly shows the fracture was dominated by excessive fiber breakage, the lack of strong CNTs interface between the fiber and matrix lead to unstoppable crack propagating. Upon growing CNTs, they act as crack stopper/deflector, as shown in Figure 6b. The crack initiated within the matrix and upon loading it gets deflected along the direction of the fibers, as shown in Figure 6b, indicating that the presence of CNTs assisted the matrix in transferring the load to the fiber. The matrix failure was also observed for the 105-micron patterned growth, as shown in Figure 6c; however, the cracks were one-sided at the interface between the fiber and matrix.

The relatively large spacing in between the grown CNT patches (105 microns) in the coarse-patterned CNTs provide more room for the fibers to be impregnated with the epoxy without interacting with the CNTs, leading to relatively weaker interfaces that have no obstacles to hinder the crack propagation. This suggests that the interspacing between the CNTs patch should be optimized. This hypothesis is supported by the fractography of the finer patterned growth, as shown in Figure 6d. The composite exhibited no cracks in between the fiber, indicating that the patterned growth induced interlocking mechanism created strong obstacles that suppressed crack formation, which led to attaining the highest strength among all the composites configurations. The inset figure in Figure 6d shows the role of the surface grown MWCNTs in blocking the crack propagation through the matrix.

### 3.3. DMTA Results

**Figure 7:**
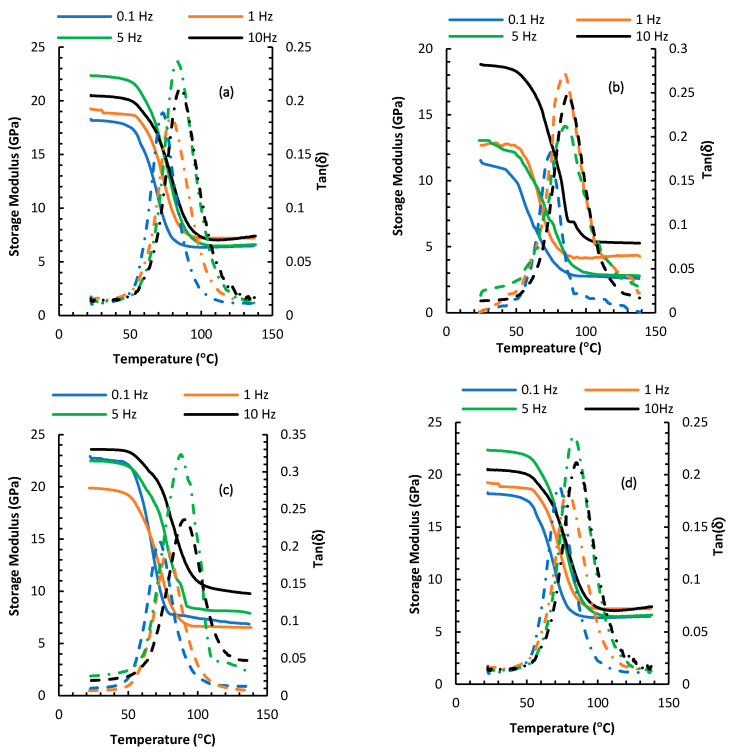
DMTA temperature sweep for composites based on (**a**) Reference, (**b**) uniform growth, (**c**) 53-micron patterned growth, and (**d**) 105-micron patterned growth fibers. The storage modulus is depicted by solid lines while tan(δ) is depicted by hatched lines.

The viscoelastic properties (storage modulus and tanδ) for the four different composites configurations from the temperature sweep at a frequency range of 0.1–10 Hz are plotted in Figure 7. As mentioned earlier, T_g_ can be found from either the first inflection point of the storage modulus curve or the peak of tan (δ) curve. The estimate for T_g_ using the two methods are tabulated in Table 1. As seen from Table 1, there are noticeable differences between the T_g_ values calculated from tan (δ) peaks, vis-a-vis those calculated from the storage modulus inflection. It was reported that a difference margin of around 25 °C can be expected between these two methods [7]. Regardless of the method of calculation of T_g_, among the different composite configurations, the 53-micron patterned composite achieved the highest value for T_g_; the change was as high as 13.15% compared to the reference composite at 10 Hz. A similar trend, but to a lesser extent, was observed for the 105-micron patterned composite. During glass transition, the polymer molecular segments absorb thermal energy and begin to move. However, the entrapment of the epoxy in between the CNTs patches diminishes some of the otherwise available space that would allow the molecular movement, hence, more energy (and a higher temperature) is needed to go around these hurdles. Interestingly, the full growth composite showed mixed results; an increase of T_g_ using the tan (δ) analysis and a decrease from the storage modulus curves was observed.

Another observation is that higher frequency yielded higher glass transition. The slight shift of the storage modulus curves to the right as the frequency increases elucidates that the glass transition depends on the frequency [46]. Glassy to rubbery transitions shift to higher temperatures at higher frequencies. The shift to higher temperatures is a direct consequence of time–temperature equivalence. As the frequency is increased, the time allowed for molecular motion is decreased, increasing the possibility of shorter timescale motions. Hence, the polymer responds more as if it were at a lower temperature than a sample run at a lower frequency but the same temperature. Consequently, higher temperatures are required for a sample to achieve an equivalent mechanical state at higher frequencies, and the transitions shift to higher temperatures [3,18,47,48].

**Figure 8:**
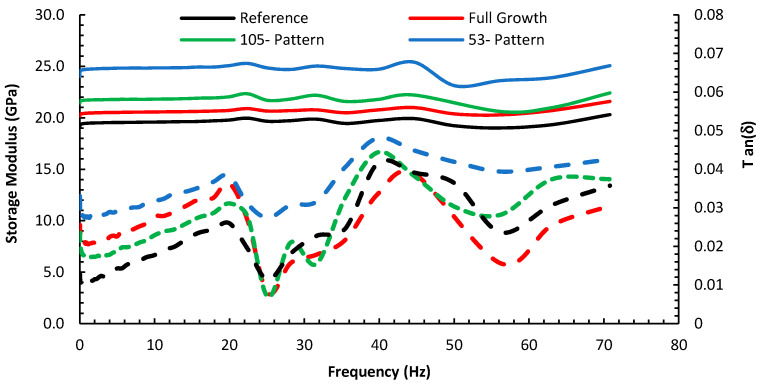
The DMTA measurements of the storage modulus and tan (δ) for the different composite’s configurations via frequency sweep at 30 °C. The storage modulus is depicted by solid lines while tan(δ) is depicted by hatched lines.

Based on the loss tangent and the storage modulus measurements from Figure 8, the growth of MWCNTs on the surface of the carbon fiber fabric promoted the mechanisms by which the composite dissipates energy leading to higher values of tan (δ) over the frequency spectra. In particular, the 53-micron patterned composite exhibited an increase in the loss tangent over the reference composite of about 46% at 20 Hz and 26% at 40 Hz, respectively. This increase is due to the presence of CNTs, resisting epoxy molecule movement, which increases friction between epoxy and the carbon fiber. While the loss tangent values are low, they agree with reported room temperature loss tangent values for carbon fiber composites; less than 0.1 in flexure mode DMTA [7]. Although, the loss tangent exhibits nonlinear frequency-dependent behavior, the same frequency-dependent pattern is observed for all composite’s configurations. The applied strain level (0.05%) is too small to activate all the mechanisms of energy dissipation via viscoelastic deformation at the CNT/epoxy interface [49,50], yet the loss tangent of the hybrid composite has noticeably increased. Inducing a patterned growth promoted further increase in tan (δ). Hence, that partial activation of frictional sliding of CNTs/polymer along with the interlocking effect on the interface region are the two possible leading mechanisms responsible for the higher loss modulus of the hybrid composites. The interface is the sole source of difference in energy dissipation in the reference composite configuration.

Figure 8 also indicates that the measured storage modulus for all composite samples exhibited a frequency-independent behavior for most of the frequency spectra. In theory, the storage moduli should be frequency-dependent for a viscous fluid or a viscoelastic material [2]. As the composite becomes more elastic (when adding elastic fillers such CNTs on carbon fibers), the viscoelastic (time-dependent) behavior diminishes, and the frequency dependence of the storage modulus vanishes. Such an observation was reported for carbon fiber composites with different fiber orientations [51] within a range of frequencies. The results for the storage moduli of different specimens, Figure 8, are qualitatively in agreement with those obtained for the modulus from the tensile tests, Figure 5c.

**Figure 9:**
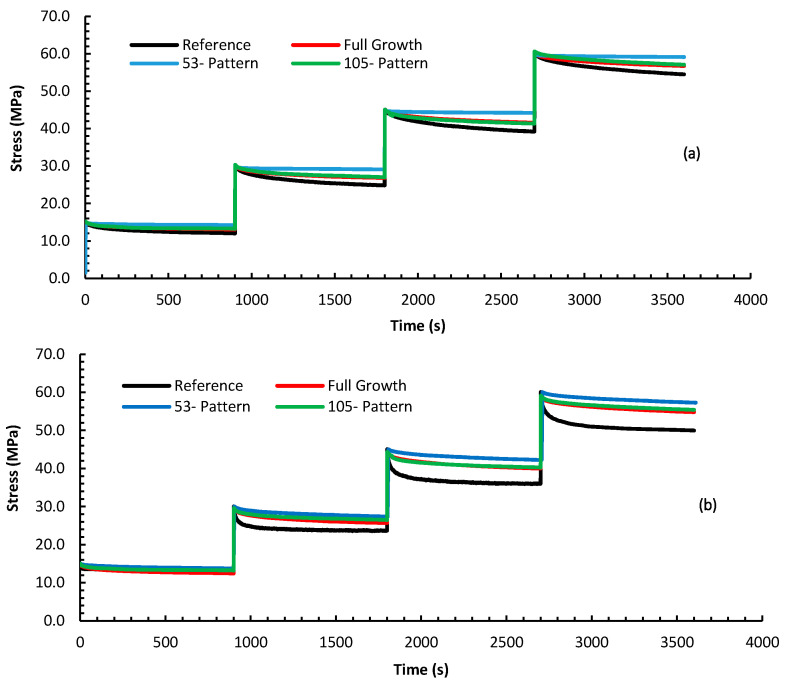

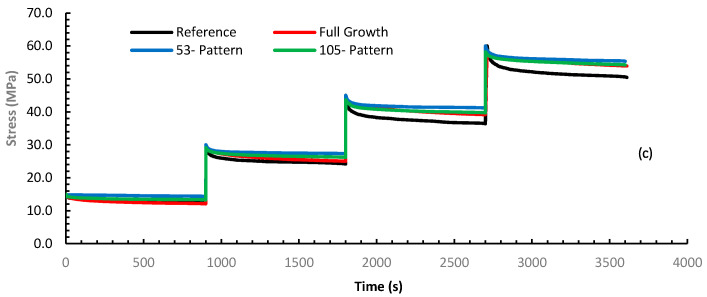
Stress relaxation curves for the different composite configurations under (**a**) 25, (**b**) 50, and (**c**) 75 °C at stress levels 15–60 MPa.

Figure 9 shows the stress relaxation at each stress level for the composites’ configurations. Because of the decaying nature of the stress/time curve during relaxation, the stress will achieve a nearly asymptotic value after enough time has elapsed. This horizontal asymptote which determines the limiting value of stress during unloading depends on the material parameters (elastic and viscoelastic parameters), the loading level and the temperature [12]. The amount of the stress decay can be considered, in lieu of the creep strain rate, as a measure of the viscoelasticity; the larger the stress reduction, the least resistant is the composite to unwanted viscoelastic behavior. Table 2 summarizes the percentage drop of the stress by normalizing the difference between the initial and final stress (i.e., stress after 30 min for each test segment) to the initial stress level. It can be inferred that, at the combination of elevated temperatures and higher stress levels, the amount of relaxation in stress increases significantly. The composite based on the reference fiber exhibited the poorest stress relaxation behavior, evident by the large stress drop percentage. This is particularly visible at 75 °C and 60 MPa where the stress dropped by 16.7% compared to 11.1% for the composite with full growth of CNTs. Imposing the topological growth patterns yielded less relaxation in the stress; 9.7% and 7.8% for the 105 and the 53 patterns, respectively. By examining the data in Table 2, the results suggest that having CNTs at the interface play a positive role in reducing the stress relaxation level. It is clear that the composite based on the 53 pattern always outperformed all the other configurations, leading to a lesser stress relaxation percentage. Again, this can be attributed to the mechanisms opposing the viscoelastic behavior such as enhanced stiffness and improved adhesion and interlocking at the epoxy/reinforcement interfaces. Additionally, one can notice the importance of having an optimal topology by examining the behavior of the full growth and the 105 pattern configurations. At several instances, the stress relaxation curves of these two configurations overlapped indicating very close performance.

Overall, one can conclude that viscoelastic behavior of FRPs is responsive to fiber/matrix interface in more than one way. The epoxy impregnation through the CNTs and its adhesion to both CNTs and carbon fiber control the strength of the interface and hence the viscoelastic behavior of the composite. Growing CNTs uniformly on the carbon fibers promote larger CNTs/matrix interface, while hindering the impregnation of the carbon fibers with the matrix. The patterned growth offers a path to resolve these conflicting factors, by allowing significant interface area between the grown CNTs and the epoxy and providing other areas at which the fibers are fully immersed in the epoxy matrix during the fabrication process.

## 4. Conclusions

In this investigation, CNTs were grown on the surface of plain-woven carbon fiber fabrics using a relatively low temperature technique, GSD. The growth was tailored into uniform, fine-, and coarse-patterned topologies. Composites were fabricated utilizing these different topologies and were tested using tensile, DMTA, and flexure stress relaxation experiments to reveal the effect of the CNTs’ topologies on different static and dynamic properties of the composites.

The results showed that composite based on fibers with fine-patterned growth of CNTs outperforms those based on uniform growth, coarse growth or no growth in terms of stiffness, tensile strength, glass transition temperature, storage modulus, damping parameter, and resistance to stress relaxation. Patterning the growth by the 53-micron pattern enhanced the strength by 19% compared to 6% for the coarser pattern of 105 micron. These results and the fractography analysis indicate that the interlocking mechanism by allowing spacing in between the CNTs patch allows for the constituents—CNTs, carbon fiber and epoxy—to adhere to each other. The composite exhibited no cracks in between the fiber, as the interlocking mechanism created strong obstacles that suppressed crack propagation which led to attaining the highest strength among all the composites’ configurations. The minute improvements by growing CNTs without a pattern is attributed to the damage induced to the fiber due to the growth temperature and the phase separation between these constituents, as reported by other investigations [29].

Depending on the frequency, utilizing full growth of CNTs yielded a drop in T_g_ at certain frequencies. In contrast, the 53-patterned growth of the CNTs shifted T_g_ upward by as high as 13.15% due to obstruction by CNTs in the movement of the epoxy due to the interlocking of the epoxy in the spaces in-between the CNTs patches. The frequency sweep DMTA test revealed that the 53-micron patterned composite exhibited an increase in the loss tangent over the reference composite of about 46% at 20 Hz. This increase is due to the presence of patterned CNTs patches hindering the epoxy molecule movement, which increases friction, leading to higher energy dissipation. The stress relaxation results highlighted the importance of using CNTs to minimize the stress decay, which leads to composite structural instability. This is particularly of importance at elevated temperatures and loading conditions. Utilizing the 53-patterned CNTs topology significantly reduced the stress decay percentage at the thermomechanical conditions of 60 MPa and 75 °C from 16.7% to 7.8%.

The results of this study emphasize that the mere growth of CNTs on carbon fibers fabric prior to composite fabrication might not yield a satisfactory performance. Rather, to realize the full benefits of hybrid composites based on multiscale reinforcements, such as CNTs and carbon fibers, the growth of CNTs should be designed into an optimal topology that enhances a plethora of the FRP properties.

## Figures and Tables

**Table 1:** Comparison of glass transition temperature for different composites’ configurations.

Configuration	Frequency (Hz)	Tg from Storage Modulus (°C)	% Change	Tg from Tan (δ) (°C)	% Change
Reference	0.1	71	0	73	0
	1	74	0	79	0
	5	75	0	82	0
	10	76	0	85	0
Full Growth	0.1	66	−7.0	77	5.47
	1	72	−2.7	84	6.33
	5	74	−1.3	85	3.65
	10	76	0	87	2.35
105-micron Pattern	0.1	72	1.4	73	0
	1	73	1.35	84	6.33
	5	81	8.0	85	3.66
	10	84	10.5	88	3.53
53-micron Pattern	0.1	72	1.4	75	2.73
	1	75	1.35	83	1.21
	5	82	9.33	88	7.31
	10	86	13.15	92	8.23

**Table 2:** Percent stress drop at each temperature and initial stress level for the different composites’ configurations.

	Initial Stress (MPa)	22 °C	50 °C	75 °C
Reference	15	14.2	14.0	13.7
	30	17.3	21.2	19.7
	45	19.3	20.2	19.1
	60	18.4	16.7	16.7
Full Growth	15	12.0	17.3	19.3
	30	10.1	14.3	16.3
	45	7.6	11.1	12.9
	60	5.5	8.6	10.2
105 Pattern	15	11.5	11.5	11.1
	30	10.0	11.6	12.7
	45	8.1	10.5	11.6
	60	5.0	7.7	9.7
53 Pattern	15	5.3	8.2	4.0
	30	3.0	8.6	3.1
	45	2.6	6.1	8.4
	60	1.6	4.5	7.8
